# 2,2-Dimethyl-*N*-(2-methyl­phenyl­sulfon­yl)propanamide

**DOI:** 10.1107/S1600536811003400

**Published:** 2011-01-29

**Authors:** K. Shakuntala, Sabine Foro, B. Thimme Gowda

**Affiliations:** a Department of Chemistry, Mangalore University, Mangalagangotri 574 199, Mangalore, India; b Institute of Materials Science, Darmstadt University of Technology, Petersenstrasse 23, D-64287 Darmstadt, Germany

## Abstract

In the title compound, C_12_H_17_NO_3_S, the amide H atom is *syn* to the *ortho*-methyl group of the benzene ring and the C—S—N—C torsion angle is −65.39 (17)°. The crystal structure features inversion-related dimers linked by pairs of N—H⋯O hydrogen bonds in which the acceptor O atom is bound to the S atom.

## Related literature

Sulfonamide drugs contain the sulfanilamide moiety (Maren, 1976 ▶). Their tendency and preferences for hydrogen bonding in the solid state can give rise to polymorphism, see: Yang & Guillory (1972 ▶); Adsmond & Grant (2001 ▶). For our studies on the effect of substituents on the crystal structures of this class of compounds, see: Gowda *et al.* (2008**a* ▶,b* ▶, 2010 ▶). 
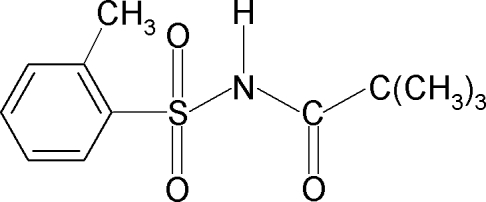


## Experimental

### 

#### Crystal data

C_12_H_17_NO_3_S
*M* *_r_* = 255.33
Monoclinic, 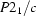

*a* = 7.3827 (6) Å
*b* = 21.986 (2) Å
*c* = 8.6060 (8) Å
β = 97.158 (9)°
*V* = 1386.0 (2) Å^3^
*Z* = 4
Cu *K*α radiation
μ = 2.06 mm^−1^
*T* = 299 K
0.30 × 0.25 × 0.25 mm

#### Data collection

Enraf–Nonius CAD-4 diffractometer
3884 measured reflections
2472 independent reflections
2202 reflections with *I* > 2σ(*I*)
*R*_int_ = 0.050
3 standard reflections every 120 min  intensity decay: 0.5%

#### Refinement

*R*[*F* ^2^ > 2σ(*F* ^2^)] = 0.037
*wR*(*F* ^2^) = 0.098
*S* = 1.05
2472 reflections
162 parameters
1 restraint
H atoms treated by a mixture of independent and constrained refinement
Δρ_max_ = 0.38 e Å^−3^
Δρ_min_ = −0.35 e Å^−3^

### 

Data collection: *CAD-4-PC* (Enraf–Nonius, 1996 ▶); cell refinement: *CAD-4-PC*; data reduction: *REDU4* (Stoe & Cie, 1987 ▶); program(s) used to solve structure: *SHELXS97* (Sheldrick, 2008 ▶); program(s) used to refine structure: *SHELXL97* (Sheldrick, 2008 ▶); molecular graphics: *PLATON* (Spek, 2009 ▶); software used to prepare material for publication: *SHELXL97*.

## Supplementary Material

Crystal structure: contains datablocks I, global. DOI: 10.1107/S1600536811003400/tk2712sup1.cif

Structure factors: contains datablocks I. DOI: 10.1107/S1600536811003400/tk2712Isup2.hkl

Additional supplementary materials:  crystallographic information; 3D view; checkCIF report

## Figures and Tables

**Table 1:** Hydrogen-bond geometry (Å, °)

*D*—H⋯*A*	*D*—H	H⋯*A*	*D*⋯*A*	*D*—H⋯*A*
N1—H1*N*⋯O1^i^	0.82 (2)	2.10 (2)	2.906 (2)	170 (2)

Symmetry code: (i) 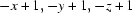.

**Fig. 1.**
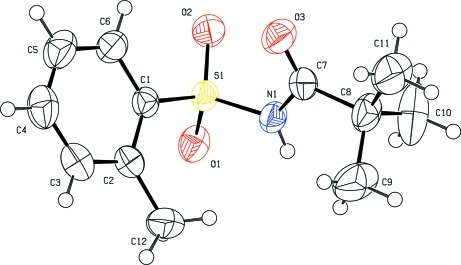
Molecular structure of (I), showing the atom- labelling scheme. Displacement ellipsoids are drawn at the 50% probability level.

**Fig. 2.**
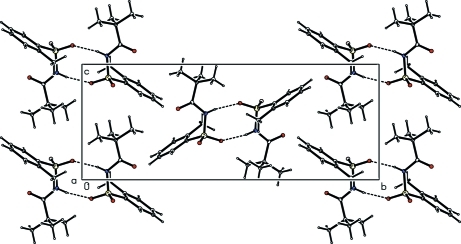
Molecular packing for (I) viewed in projection down the a axis. Hydrogen bonds are shown as dashed lines.

## supplementary crystallographic information

### Comment

The molecular structures of sulfonamide drugs contain the sulfanilamide moiety (Maren, 1976), and their propensity for hydrogen bonding in the solid state, due to the presence of various hydrogen bond donors and acceptors, can give rise to polymorphism (Yang & Guillory, 1972; Adsmond & Grant, 2001). Hence, the nature and position of substituents play a significant role on the crystal structures of *N*-(aryl)sulfonoamides. As a part of a study of the substituent effects on the crystal structures of this class of compounds (Gowda *et al.*, 2008**a*,b*, 2010), the structure of *N*-(2-methylphenylsulfonyl)-2,2,2- trimethylacetamide (I) has been determined.

The N—H and C=O bonds are *anti* to each other (Fig. 1), as observed in each of *N*-(phenylsulfonyl)acetamide (II) (Gowda *et al.*, 2010), *N*-(phenylsulfonyl)-2,2,2-trimethylacetamide (III) (Gowda *et al.*, 2008*b*) and *N*-(4-methylphenylsulfonyl)-2,2,2-trimethylacetamide (IV) (Gowda *et al.*, 2008*a*). Further, the amide hydrogen is *syn* to the *ortho*-methyl group in the benzene ring. The molecule in (I) is bent at the *S*-atom with the C1—S1—N1—C7 torsion angle being -65.39 (17)°, compared to the values of -58.8 (4)° in (II), -64.5 (3)° in (III) and -68.2 (2)° in (IV).

In the crystal structure, the pairs of intermolecular N–H···O hydrogen bonds (Table 1) link inversion-related molecules into dimeric aggregates where the acceptor O atom is bound to the S atom; Fig. 2.

### Experimental

Compound (I) was prepared by refluxing 2-methylbenzenesulfonamide (0.10 mole) with an excess of pivalyl chloride (0.20 mole) for about an hour on a water bath. The reaction mixture was cooled and poured into ice cold water. The resulting solid was separated, washed thoroughly with water and dissolved in warm dilute sodium hydrogen carbonate solution.Compound (I) was reprecipitated by acidifying the filtered solution with glacial acetic acid. It was filtered, dried and recrystallized from ethanol. Prism like red crystals were obtained from a slow evaporation of an ethanolic solution of (I).

### Refinement

The amide-H atom was located in a difference map and refined with the distance restraint N—H = 0.86 (2) Å. The other H atoms were positioned with idealized geometry using a riding model with C—H = 0.93–0.96 Å. All H atoms were refined with isotropic displacement parameters (set to 1.2 times of the *U*_eq_ of the parent atom).

### Crystal data

**Table:**

C_12_H_17_NO_3_S	*F*(000) = 544
*M**_r_* = 255.33	*D*_x_ = 1.224 Mg m^−^^3^
Monoclinic, *P*2_1_/*c*	Cu *K*α radiation, λ = 1.54180 Å
Hall symbol: -P 2ybc	Cell parameters from 25 reflections
*a* = 7.3827 (6) Å	θ = 6.0–21.6°
*b* = 21.986 (2) Å	µ = 2.06 mm^−^^1^
*c* = 8.6060 (8) Å	*T* = 299 K
β = 97.158 (9)°	Prism, red
*V* = 1386.0 (2) Å^3^	0.30 × 0.25 × 0.25 mm
*Z* = 4	

### Data collection

**Table:**

Enraf–Nonius CAD-4 diffractometer	*R*_int_ = 0.050
Radiation source: fine-focus sealed tube	θ_max_ = 67.0°, θ_min_ = 4.0°
graphite	*h* = −8→4
ω/2θ scans	*k* = −26→0
3884 measured reflections	*l* = −10→10
2472 independent reflections	3 standard reflections every 120 min
2202 reflections with *I* > 2σ(*I*)	intensity decay: 0.5%

### Refinement

**Table:**

Refinement on *F*^2^	Secondary atom site location: difference Fourier map
Least-squares matrix: full	Hydrogen site location: inferred from neighbouring sites
*R*[*F*^2^ > 2σ(*F*^2^)] = 0.037	H atoms treated by a mixture of independent and constrained refinement
*wR*(*F*^2^) = 0.098	*w* = 1/[σ^2^(*F*_o_^2^) + (0.0444*P*)^2^ + 0.5143*P*] where *P* = (*F*_o_^2^ + 2*F*_c_^2^)/3
*S* = 1.05	(Δ/σ)_max_ = 0.001
2472 reflections	Δρ_max_ = 0.38 e Å^−^^3^
162 parameters	Δρ_min_ = −0.35 e Å^−^^3^
1 restraint	Extinction correction: *SHELXL97* (Sheldrick, 2008), Fc^*^=kFc[1+0.001xFc^2^λ^3^/sin(2θ)]^-1/4^
Primary atom site location: structure-invariant direct methods	Extinction coefficient: 0.0174 (8)

### Special details

**Table:**

Geometry. All e.s.d.'s (except the e.s.d. in the dihedral angle between two l.s. planes) are estimated using the full covariance matrix. The cell e.s.d.'s are taken into account individually in the estimation of e.s.d.'s in distances, angles and torsion angles; correlations between e.s.d.'s in cell parameters are only used when they are defined by crystal symmetry. An approximate (isotropic) treatment of cell e.s.d.'s is used for estimating e.s.d.'s involving l.s. planes.
Refinement. Refinement of *F*^2^ against ALL reflections. The weighted *R*-factor *wR* and goodness of fit *S* are based on *F*^2^, conventional *R*-factors *R* are based on *F*, with *F* set to zero for negative *F*^2^. The threshold expression of *F*^2^ > σ(*F*^2^) is used only for calculating *R*-factors(gt) *etc*. and is not relevant to the choice of reflections for refinement. *R*-factors based on *F*^2^ are statistically about twice as large as those based on *F*, and *R*- factors based on ALL data will be even larger.

### Fractional atomic coordinates and isotropic or equivalent isotropic displacement parameters (Å^2^)

**Table:**

	*x*	*y*	*z*	*U*_iso_*/*U*_eq_	
C1	0.5655 (2)	0.64776 (8)	0.66047 (17)	0.0386 (4)	
C2	0.7426 (2)	0.64421 (9)	0.6175 (2)	0.0460 (4)	
C3	0.8623 (3)	0.69061 (10)	0.6714 (2)	0.0574 (5)	
H3	0.9803	0.6902	0.6443	0.069*	
C4	0.8114 (3)	0.73713 (10)	0.7636 (3)	0.0643 (6)	
H4	0.8957	0.7669	0.7998	0.077*	
C5	0.6366 (3)	0.73991 (10)	0.8024 (3)	0.0637 (6)	
H5	0.6026	0.7715	0.8644	0.076*	
C6	0.5122 (3)	0.69569 (9)	0.7493 (2)	0.0495 (4)	
H6	0.3928	0.6979	0.7728	0.059*	
C7	0.2796 (2)	0.62930 (9)	0.32969 (19)	0.0452 (4)	
C8	0.2533 (3)	0.61352 (10)	0.1557 (2)	0.0574 (5)	
C9	0.4402 (4)	0.60025 (15)	0.1019 (3)	0.0859 (9)	
H9A	0.4938	0.5654	0.1569	0.103*	
H9B	0.5187	0.6348	0.1237	0.103*	
H9C	0.4248	0.5922	−0.0087	0.103*	
C10	0.1352 (5)	0.55743 (18)	0.1291 (3)	0.1121 (12)	
H10A	0.0191	0.5649	0.1648	0.135*	
H10B	0.1942	0.5239	0.1861	0.135*	
H10C	0.1173	0.5479	0.0193	0.135*	
C11	0.1705 (5)	0.66816 (14)	0.0647 (3)	0.0888 (9)	
H11A	0.2493	0.7027	0.0855	0.107*	
H11B	0.0532	0.6770	0.0965	0.107*	
H11C	0.1566	0.6592	−0.0453	0.107*	
C12	0.8070 (3)	0.59462 (12)	0.5168 (3)	0.0670 (6)	
H12A	0.7316	0.5940	0.4175	0.080*	
H12B	0.7990	0.5561	0.5679	0.080*	
H12C	0.9314	0.6022	0.5008	0.080*	
N1	0.3642 (2)	0.58401 (7)	0.42600 (16)	0.0439 (4)	
H1N	0.403 (3)	0.5529 (8)	0.390 (2)	0.053*	
O1	0.49106 (18)	0.53203 (6)	0.66336 (13)	0.0508 (3)	
O2	0.24290 (17)	0.60428 (7)	0.68099 (15)	0.0536 (4)	
O3	0.2347 (2)	0.67637 (7)	0.38512 (15)	0.0604 (4)	
S1	0.40583 (5)	0.588952 (19)	0.61796 (4)	0.03927 (17)	

### Atomic displacement parameters (Å^2^)

**Table:**

	*U*^11^	*U*^22^	*U*^33^	*U*^12^	*U*^13^	*U*^23^
C1	0.0424 (9)	0.0401 (9)	0.0319 (7)	0.0023 (7)	−0.0013 (6)	0.0018 (6)
C2	0.0418 (9)	0.0505 (10)	0.0441 (9)	0.0040 (8)	−0.0020 (7)	0.0020 (8)
C3	0.0455 (10)	0.0631 (13)	0.0613 (12)	−0.0043 (9)	−0.0029 (9)	0.0057 (9)
C4	0.0690 (14)	0.0542 (12)	0.0650 (13)	−0.0143 (10)	−0.0107 (10)	−0.0032 (10)
C5	0.0817 (15)	0.0485 (12)	0.0588 (12)	−0.0006 (10)	0.0006 (11)	−0.0143 (9)
C6	0.0556 (10)	0.0488 (10)	0.0438 (9)	0.0043 (8)	0.0049 (8)	−0.0049 (8)
C7	0.0458 (9)	0.0525 (11)	0.0360 (8)	0.0026 (8)	−0.0005 (7)	0.0001 (7)
C8	0.0759 (13)	0.0610 (13)	0.0325 (9)	0.0066 (11)	−0.0040 (8)	−0.0011 (8)
C9	0.113 (2)	0.102 (2)	0.0456 (12)	0.0371 (17)	0.0222 (13)	0.0108 (12)
C10	0.145 (3)	0.113 (3)	0.0665 (16)	−0.038 (2)	−0.0314 (17)	−0.0137 (16)
C11	0.122 (2)	0.098 (2)	0.0431 (11)	0.0481 (18)	−0.0054 (12)	0.0072 (11)
C12	0.0462 (11)	0.0754 (16)	0.0807 (15)	0.0058 (10)	0.0127 (10)	−0.0192 (12)
N1	0.0523 (9)	0.0455 (9)	0.0321 (7)	0.0068 (7)	−0.0017 (6)	−0.0044 (6)
O1	0.0696 (8)	0.0416 (7)	0.0393 (6)	0.0031 (6)	−0.0009 (6)	0.0037 (5)
O2	0.0457 (7)	0.0712 (9)	0.0455 (7)	−0.0024 (6)	0.0112 (5)	−0.0053 (6)
O3	0.0777 (10)	0.0550 (8)	0.0466 (7)	0.0184 (7)	0.0007 (6)	−0.0044 (6)
S1	0.0434 (3)	0.0433 (3)	0.0304 (2)	0.00067 (17)	0.00183 (16)	−0.00052 (15)

### Geometric parameters (Å, °)

**Table:**

C1—C6	1.387 (3)	C8—C9	1.537 (3)
C1—C2	1.405 (2)	C9—H9A	0.9600
C1—S1	1.7570 (17)	C9—H9B	0.9600
C2—C3	1.391 (3)	C9—H9C	0.9600
C2—C12	1.506 (3)	C10—H10A	0.9600
C3—C4	1.375 (3)	C10—H10B	0.9600
C3—H3	0.9300	C10—H10C	0.9600
C4—C5	1.374 (3)	C11—H11A	0.9600
C4—H4	0.9300	C11—H11B	0.9600
C5—C6	1.376 (3)	C11—H11C	0.9600
C5—H5	0.9300	C12—H12A	0.9600
C6—H6	0.9300	C12—H12B	0.9600
C7—O3	1.203 (2)	C12—H12C	0.9600
C7—N1	1.393 (2)	N1—S1	1.6459 (14)
C7—C8	1.526 (2)	N1—H1N	0.817 (16)
C8—C10	1.511 (4)	O1—S1	1.4330 (13)
C8—C11	1.520 (3)	O2—S1	1.4204 (13)
			
C6—C1—C2	121.63 (17)	C8—C9—H9C	109.5
C6—C1—S1	116.41 (14)	H9A—C9—H9C	109.5
C2—C1—S1	121.78 (14)	H9B—C9—H9C	109.5
C3—C2—C1	116.41 (18)	C8—C10—H10A	109.5
C3—C2—C12	119.35 (18)	C8—C10—H10B	109.5
C1—C2—C12	124.24 (18)	H10A—C10—H10B	109.5
C4—C3—C2	122.0 (2)	C8—C10—H10C	109.5
C4—C3—H3	119.0	H10A—C10—H10C	109.5
C2—C3—H3	119.0	H10B—C10—H10C	109.5
C5—C4—C3	120.4 (2)	C8—C11—H11A	109.5
C5—C4—H4	119.8	C8—C11—H11B	109.5
C3—C4—H4	119.8	H11A—C11—H11B	109.5
C4—C5—C6	119.7 (2)	C8—C11—H11C	109.5
C4—C5—H5	120.1	H11A—C11—H11C	109.5
C6—C5—H5	120.1	H11B—C11—H11C	109.5
C5—C6—C1	119.75 (19)	C2—C12—H12A	109.5
C5—C6—H6	120.1	C2—C12—H12B	109.5
C1—C6—H6	120.1	H12A—C12—H12B	109.5
O3—C7—N1	120.29 (16)	C2—C12—H12C	109.5
O3—C7—C8	125.23 (17)	H12A—C12—H12C	109.5
N1—C7—C8	114.48 (16)	H12B—C12—H12C	109.5
C10—C8—C11	112.3 (2)	C7—N1—S1	124.33 (13)
C10—C8—C7	109.58 (19)	C7—N1—H1N	121.7 (15)
C11—C8—C7	108.65 (18)	S1—N1—H1N	113.9 (15)
C10—C8—C9	108.8 (3)	O2—S1—O1	117.84 (8)
C11—C8—C9	108.3 (2)	O2—S1—N1	109.75 (8)
C7—C8—C9	109.25 (17)	O1—S1—N1	103.69 (7)
C8—C9—H9A	109.5	O2—S1—C1	108.90 (8)
C8—C9—H9B	109.5	O1—S1—C1	108.99 (8)
H9A—C9—H9B	109.5	N1—S1—C1	107.11 (8)
			
C6—C1—C2—C3	1.3 (3)	N1—C7—C8—C11	−176.7 (2)
S1—C1—C2—C3	−173.65 (13)	O3—C7—C8—C9	120.8 (2)
C6—C1—C2—C12	−177.79 (19)	N1—C7—C8—C9	−58.8 (2)
S1—C1—C2—C12	7.3 (3)	O3—C7—N1—S1	1.5 (3)
C1—C2—C3—C4	0.8 (3)	C8—C7—N1—S1	−178.99 (14)
C12—C2—C3—C4	179.9 (2)	C7—N1—S1—O2	52.69 (18)
C2—C3—C4—C5	−1.6 (3)	C7—N1—S1—O1	179.43 (15)
C3—C4—C5—C6	0.3 (3)	C7—N1—S1—C1	−65.39 (17)
C4—C5—C6—C1	1.8 (3)	C6—C1—S1—O2	2.45 (16)
C2—C1—C6—C5	−2.6 (3)	C2—C1—S1—O2	177.61 (13)
S1—C1—C6—C5	172.59 (15)	C6—C1—S1—O1	−127.33 (13)
O3—C7—C8—C10	−120.1 (3)	C2—C1—S1—O1	47.82 (15)
N1—C7—C8—C10	60.3 (3)	C6—C1—S1—N1	121.08 (14)
O3—C7—C8—C11	2.9 (3)	C2—C1—S1—N1	−63.76 (15)

### Hydrogen-bond geometry (Å, °)

**Table:**

*D*—H···*A*	*D*—H	H···*A*	*D*···*A*	*D*—H···*A*
N1—H1N···O1^i^	0.82 (2)	2.10 (2)	2.906 (2)	170 (2)

Symmetry codes: (i) −*x*+1, −*y*+1, −*z*+1.
